# Supplementary material for: Are carbon emissions trading and green financial instruments synergistic? -Comprehensive quantitative research based on content analysis
Source: PLoS One. 2024 Mar 7;19(3):e0298601. doi: 10.1371/journal.pone.0298601 (PMC10919601; doi:10.1371/journal.pone.0298601)
Supplement: S1 Data — (ZIP) [file pone.0298601.s001.zip › Data/地级市Z_F工作报告.zip!/║■─╧/│ñ╔│╩╨/└┤╫╘╣1⁄2╓┌║┼í╛┬φ┐╦╩2╛▌═°í┐_2006.docx]

各位代表：

　　现在，我代表市人民政府向大会作关于长沙市国民经济和社会发展第十一个五年规划纲要的报告，请大家连同《规划纲要》（草案）一并审议，并请各位政协委员和其他列席人员提出意见。

　　一、关于“十五”计划的执行情况

　　新世纪之初的五年，全市人民在市委的正确领导下，以邓小平理论和“三个代表”重要思想为指导，用科学发展观统领经济社会发展全局，锐意进取，自我加压，团结实干，战胜了各种困难与挑战，取得了令人鼓舞和欣慰的重大成就，全市呈现出蓬勃发展、文明进步、安定和谐的崭新局面，“十五”成为长沙历史上发展最快最好的时期。

　　（一）始终坚持以发展为第一要务，突出兴工强市战略，巩固和加强农业基础地位，保持第三产业发展优势，经济运行质量和效益整体提高，步入了全面协调可持续发展轨道。国民经济快速增长，2005年全市实现地区生产总值1517亿元（预计数，下同），同比增长15%，年均增长14.8%，五年翻了一番，占全省比重达23.3%，在全国省会城市中排位前移2位。工业经济规模实现翻番目标，2005年完成工业总产值1285亿元，工业增加值年均增长16.5%。认真落实中央一号文件精神，农业发展后劲明显增强，近两年免征农业税和除烟叶以外的农业特产税2.47亿元，落实各项农业政策直补资金1.68亿元，2005年实现农业总产值190亿元，年均增长5.5%。第三产业稳健增长，2005年实现社会消费品零售总额610亿元，年均增长14.7%，增幅位居全国前列。地方财政收入提前两年实现“十五”计划目标，2005年完成地方财政收入139.38亿元，五年增长了3.94倍，年均增长31.53%；完成全社会固定资产投资915亿元，年均增长35.2%；城乡居民人均可支配收入分别达12300元和4600元，年均增长10.3%和9.4%。长沙综合实力位居全国省会城市第9位，已经在一个新的起点上向实现全面小康社会的宏伟目标迈进！

　　（二）始终坚持以结构调整为主线，优化产业、所有制和投资结构，发展壮大开放型经济和非公有制经济，经济自主增长能力不断增强，结构竞争力显著提升。产业结构更趋合理，三次产业比由2000年的10.3:38.9:50.8调整为2005年的7.6:44.1:48.3。新型工业化进程加速推进，2005年工业增加值占GDP比重达31.6%，高新技术产业总产值达580亿元。农业产业化程度不断提高，全市农业产业化龙头企业达1825家，2005年实现农产品加工销售收入190亿元。商贸优势更加凸现，现代服务业快速发展，2005年实现旅游总收入165亿元，保费收入27.1亿元，动漫原创产品产值占全国二分之一。多种所有制经济竞相发展，国企改革走在全国前列，五年实施企业改制173户；2005年非公有制经济占全市经济总量比重达50.2%。开放型经济不断壮大，2005年全市引进外来资金430亿元，其中实际到位外资9亿美元；五年累计实际利用外资25亿美元，实现进出口总额106亿美元，年均分别增长38.4%和9.9%；赴境外投资企业累计达48家；已有88家世界500强企业在长沙投资办厂或设立办事机构。2005年全市非国有投资占全社会固定资产投资的70.2%，比“九五”末提高25.2个百分点。在全国50座主要城市竞争力排名中，长沙结构竞争力位居第3位。

　　（三）始终坚持以规划为龙头，城市建设与管理并重，注重拓展新城与保护历史文化名城相结合，走城市经营之路，城乡面貌发生深刻变化。加快推进城市化进程，2005年建成区面积达153平方公里，市区人口达206万人，城市化率达53%，五年提高8.4个百分点。新一轮城市总体规划修编顺利完成，城市规划能力居全国第7位。五年投入城市建设资金482亿元，完成重点工程建设200项；改造建设城市主次干道158条，新增城市道路325公里，“六桥三环”的城市交通格局基本形成。实施了棚户区、背街小巷及历史区街的改造、修缮和维护工程，历史文化名城保护取得新进展。建设了以湘江风光带、新世纪体育文化中心、杜甫江阁、简牍博物馆等为标志的一系列文化体育生态景观设施。深化城市公用事业改革，城市综合配套功能不断增强，完成了天燃气入长工程，启用了桥驿大型垃圾无害化处理场，建成了八水厂、第一污水处理厂，城市公交、供水能力位居全国前列。全面实行城管综合执法，大力开展城市环境综合整治，人居环境大为改善，五年净增城市园林绿地面积1637公顷。2005年人均公共绿地面积达7.7平方米；人均住宅建筑面积达27.2平方米；空气质量优良率达67.1%，五年提高20.1个百分点；8个区县（市）通过国家生态示范区验收。五年投入小城镇建设资金233.6亿元。改造县乡公路2000公里。完成水利工程投资44.5亿元，建成了十大城市防洪提质工程，连续四年夺取全省“水利建设芙蓉杯”一等奖。启动了18个省级农村小康示范村建设。日益变化的农村新面貌、绮丽秀美的文化生态型山水城市的独特风姿，使长沙这座历史文化名城焕发了新的时代光彩!

　　（四）始终坚持以人为本，全面推进各项社会事业发展，建立健全社会保障体系，使人民群众根本利益得到切实实现、维护和发展。近两年，全市共投入资金60多亿元，实施了省市八件实事工程，解决了一批与群众生产生活息息相关的热点难点问题。2005年市本级新增财力的71%投入到民本民生。五年累计新增就业35万人，城镇登记失业率为4.2%，近三年转移农村劳动力231万人，我市被国务院授予“全国再就业先进集体”。社会保障体系日趋完善，全市养老、医疗、失业、工伤、生育五种保险参保人数分别达89万人、81万人、56万人、38万人、47万人。在中西部地区率先启动农村低保工作。社会救助体系初步形成，建立了教育、医疗和住房等各类救助基金。农村中小学D级危房改造基本完成，对义务教育阶段家庭贫困学生实行“两免一补”，长沙大学顺利改办升格为普通本科高校。五年申请专利12016件，我市被评为全国首批区域技术创新示范城市和全国科技进步先进城市。创新人才评价和柔性流动机制，在中西部省会城市率先推行人才居住证制度。市公共卫生救治中心、疾病预防控制中心、医疗紧急救援体系建设基本完成，新型农村合作医疗试点工作顺利推进，有效防控了非典和人禽流感等重大疫情。食品药品监管力度加大。11种产品被评为中国驰名商标或中国名牌产品。国防教育和民兵预备役建设不断加强。5件作品获全国“五个一工程”奖或国家政府奖。我市被授予全国群众体育先进单位、全国国土资源管理系统先进集体、全国质量兴市先进市，三度荣获全国双拥模范市，连续四年被评为全省唯一的计划生育工作模范市。统计、审计、物价、税务、海关、人防、民族宗教、台办、新闻出版、档案、市志、消防、电力、气象、地震、水文、残联、侨联、关协、老龄委等工作都取得新的进步。

　　（五）始终坚持三个文明协调发展，切实加强民主法制建设，努力创建文明长沙、平安长沙和诚信长沙，不断扩大对外交流与合作，使长沙的知名度和整体形象日益提升。政治文明建设取得新成果，坚持执行人民代表大会及其常委会决议并自觉接受监督，坚持重大事项事先与民主党派、无党派人士协商。五年提请市人大常委会审议地方性法规（草案）23件，制定政府规章36件。办复人大代表议案、建议及政协委员提案2749件。行政村由2000年的2786个合并为1276个，城市社区居委会由604个调整为385个，并推行了直选，我市被授予全国首批社区建设示范市。政务、村务和厂务公开向纵深拓展。“四五”普法成效明显。建成宣传文化法制中心（站）478个。推进平安长沙建设，完善社会治安防控网络，建立集中处理信访突出问题及群体性事件联席会议制度，构建安全生产长效机制，人民群众安全感和满意度不断增强，我市被授予全国社会治安综合治理优秀地市，天心区获全国社会治安综合治理先进集体称号。精神文明建设取得新成绩，两度荣获全国文明城市创建工作先进市。诚信长沙建设全面启动，金融安全区创建取得良好效果。对外交流与合作更加广泛，与国内外11个城市缔结为友好城市或友好经贸合作城市。成功举办了第五届全国城市运动会、第11届世界旅游小姐年度皇后总决赛、国际友好经贸合作洽谈会等重大赛事和节会活动，长沙影响力日益扩大。

　　（六）始终坚持推进政府职能转变，深化政府机构改革和制度创新，强化社会管理和公共服务职能，政府领导发展的能力显著增强。五年来，我们围绕建设勤政廉洁、务实高效和可问责的服务型政府目标，不断创新运行机制，坚持依法从严治政，政府行政能力明显增强。积极推进政府机构和事业单位改革，完善政府决策机制，制定并实施了政府工作规则和重大事项公示、政策性减免规费集体审批、价格决策听证等制度。深化行政审批制度改革，清理行政审批项目1076项，向社会公布了市级行政许可实施项目目录；加速电子政务建设，建立各级政务服务中心，全面推行行政许可集中办理、联合办理和收费公示制；建立政府新闻发言人制度，率先推行行政问责制度。加强政府宏观调控能力，保持价格总体水平基本稳定，完善要素市场体系，规范市场经济秩序，优化投资环境；开展了全市第一次经济普查，建立经济预警预报体系；改革投融资体制，设立政府偿债风险准备金，政府经济调节职能实现有效转变。全面推行财政体制改革，五年市本级审减基建预决算70亿元，全市政府采购节减11亿元。树立良好政风，提倡勤俭节约办事情，不断克服形式主义和虚报浮夸的不良风气，各级政府务实求真的工作作风、卓有成效的工作业绩不断赢得了人民群众的拥护和赞许。

　　各位代表，“十五”期间所取得的成绩与进步，是全市人民辛勤劳动、团结奋斗的结果，是社会各界协同配合、大力支持的结果。在此，我代表市人民政府，向全市人民、驻长解放军、武警官兵和政法干警，向各民主党派、工商联、无党派人士和各人民团体，向所有关心和支持长沙建设与发展的各界朋友表示衷心的感谢，并致以崇高的敬意！

　　回顾过去的五年，我们清醒地认识到仍然存在许多突出的矛盾和问题：一是经济增长方式还需进一步转变，经济外向度还很低，资源利用率不高，经济增长缺乏大产业、大企业支撑；二是农民增收基础不稳，农村基础设施和社会事业基础比较薄弱，统筹城乡发展的任务十分艰巨；三是基层基础工作存在薄弱环节，新形势下社区管理和村民自治工作有待进一步加强和创新；四是构建和谐社会面临诸多挑战，就业压力比较大，低收入群体生活比较困难，安全生产、社会治安和环境保护等问题依然突出。上述问题，我们将在改革和发展中采取更为有效的措施认真加以解决。

　　二、“十一五”时期经济社会发展的主要目标和展望

　　“十一五”时期要努力实现以下目标：地区生产总值年均增长13%，地方财政收入年均增长15%，全社会固定资产投资年均增长15%，社会消费品零售总额年均增长14%。非公有制经济占地区生产总值比重达65%左右，高新技术产业增加值占地区生产总值比重达20%，单位地区生产总值能耗降低15%左右，五年累计直接利用外资56亿美元以上。城乡居民人均可支配收入年均分别增长9%和8%，城镇登记失业率控制在5%以内，人口自然增长率控制在5‰以内，城市空气环境污染指数年均值保持在94以内，城市污水处理率达到80%。为确保实现上述目标，必须牢固树立三个理念：

　　一是坚持科学发展的理念。更加注重经济增长的质量和效益，增强经济自主增长能力，保持经济平稳较快增长；更加注重经济结构的战略性调整，切实转变经济增长方式；更加注重统筹城乡协调发展，构建以城带乡、以工促农的长效机制；更加注重深化改革和扩大开放，建立和完善落实科学发展观的保障体系。

　　二是坚持创新发展的理念。把创新作为经济社会发展的不竭动力，不断推进观念创新、制度创新，破除一切阻碍和影响发展的体制性障碍；推进技术创新，增强原始创新、集成创新和引进消化吸收再创新能力，提升产业核心竞争力；推进教育创新、文化创新，在城市人文精神和城市形象品质提升等方面为发展提供智力支撑和内在推动力。

　　三是坚持和谐发展的理念。突出统筹经济社会协调发展，促进社会全面进步；突出以人为本，促进人的全面发展；突出解决关系群众切身利益的问题，维护社会公平与正义；突出处理好改革发展稳定的关系，确保社会大局稳定和长治久安；突出做好人口、资源与环境保护工作，促进人与自然和谐发展。

　　为全面完成“十一五”规划目标，我们要着力抓好以下工作：一是着力转变经济增长方式，继续推进新型工业化进程，大力发展现代农业和新型服务业，保持经济又快又好发展。围绕增强产业核心竞争力，大力开发具有自主知识产权的关键技术和核心技术。坚持节约资源、保护环境的基本国策，促进产业发展由粗放型增长向集约型增长转变。重点制定实施产业用地、水耗、能耗、环保、就业容量等方面的标准，按照循环经济模式加强对工业园区和主导产业的建设改造。大力发展高技术含量、高附加值、高效益的现代产业。重点培育工程机械、汽车制造、家电等优势产业集群和电子信息、新材料、生物医药等新兴产业；加快发展信息服务、现代物流、金融证券、会展等新型服务业；大力发展优质水稻、优质茶叶、花卉苗木、优质水产等优势农业；发展壮大媒体传播、出版发行、动漫游戏、文化旅游、娱乐休闲等文化产业。二是着力推进城乡统筹发展，加大对农村经济和社会事业发展的投入，建设社会主义新农村。加大公共财政对农村投入的比重，统筹规划建设城乡公共设施、居住社区、产业布局和生态环境。加快小城镇建设，全面启动村庄整治工程，重点加快乡村公路、安全卫生民居、农村新能源开发等农村基础设施建设。发展壮大县域经济，加快农业产业基地建设，大力发展农产品加工企业，加大农村水利、电网改造、沼气建设力度。创新劳务经济发展新机制。加快农村社会事业发展，不断完善农村文化设施，切实加强对农民的法律、科学文化和专业技能培训。全面推行新型农村合作医疗制度，建立大病医疗救助等农村社会救助体系。探索建立新型农村社会养老制度。三是着力实施城市可持续发展战略，加强基础设施建设和生态环境保护，提升城市整体功能与品位。重点开发建设“一江两岸”、“四大组团”、河西新城、星马新城、城南新区，推动星沙、望城与主城区的全面对接和深度融合，加快浏阳、宁乡两个卫星城市建设。重点抓好霞凝港三期工程、火车南北货站迁建工程、引水及水质环境工程、黄花机场扩建、城市轨道交通、城市防洪圈和城市生态圈等重大项目建设。切实保护利用城市绿地、山体、水体等自然资源，重点保护好岳麓山、湘江、浏阳河等城市生态功能区域，加快湘江滨水区改造建设。全面开展清洁能源行动，创建全国环保模范城市。四是着力深化体制改革，努力扩大区域合作、对外开放和对外交流，提高外向型经济的层次和水平。深化行政管理体制改革，加快构建完善的公共财政体系，分类推进事业单位改革。深化经济体制改革，加快建立现代企业制度和现代产权制度，健全国有资产管理、监督和营运体系，加快培育和发展要素市场，大力发展非公有制经济。全面深化农村综合配套改革。推进科技、教育、文化、卫生、体育等社会事业改革。深入实施开放带动战略，重点引进战略投资者，积极拓展对外贸易经济合作，发挥长沙在长株潭城市群中的核心增长极作用，推动与武汉协作区、泛珠三角和中西部各省市的互动协作。五是着力加强民主法制和精神文明建设，推进社会事业全面进步，努力构建和谐长沙。大力发展社会主义民主，加强基层民主政治建设，加快行政管理和重大决策的科学化、民主化和法制化进程。深化文明创建，努力争创全国文明城市。加强诚信长沙建设，完善社会信用体系框架。努力扩大就业，积极发展就业容量大的劳动密集型企业、中小企业和服务业，加大对失业和特困群体的就业援助力度。健全城乡社会保障体系，加快推进社会福利、社会慈善、优待抚恤事业发展。高度重视老龄工作和未成年人教育工作，注重建设服务老年人和青少年活动的场所与设施。切实提高义务教育水平，大力发展职业教育，全面启动标准化学校建设工程，逐步推行义务教育免费制度、贫困学生接受高等教育资助制度。开发人才资源，发展各类人才市场。加强疾病预防控制体系和医疗救助体系建设，重点加强农村卫生资源配置。深化平安长沙创建工作，构建完备的社会治安、安全生产、公共卫生安全、自然灾害预警预防体系和处置突发事件应急机制。开展“五五”普法。切实抓好信息化条件下的全民国防教育、民兵预备役、国防后备力量和人民防空建设。努力构建民主法治、公平正义、文明诚信、团结友爱、社会进步的和谐长沙！

　　各位代表！“十一五”时期，将是我市经济和社会发展迈上新台阶、综合实力更加增强的重要时期；是把长沙建设成为经济持续发展、生态环境优美、社会文明进步、人民生活富裕的全面小康社会的关键时期！通过全市人民的不懈努力，我们有理由相信，到“十一五”期末，长沙将呈现出更加喜人的崭新景象：

　　区域性中心城市的作用将更加显现。全市城市化率达61%,都市区城市建设用地达230平方公里，城市人口达245万人。地区生产总值达2800亿元左右，约占全省28%，人均GDP达到40000元以上，地方财政收入接近300亿元。基本建成区域性先进制造业中心，成为中部地区具有鲜明特色和重要地位的新型工业基地，全部工业总产值突破3000亿元，工业增加值达980亿元。基本建成区域性现代服务业中心，形成增加值高、辐射力强、就业面广的现代服务业体系，第三产业增加值达1330亿元左右。基本建成区域性优质农业中心，优质高效农业发展走在中部前列，农产品加工业总产值达700亿元。基本建成区域性文化产业中心，文化产业增加值占地区生产总值比重达10%以上，形成一批在国内外具有广泛影响的文化产业知名品牌。社会主义新农村建设将展现新面貌。县乡公路全面实现硬化改造，通村公路硬化率90%以上。标准化学校比例大幅提高，实现农村义务教育全免费。完成标准化乡镇卫生院建设和村卫生所改造。新型农村合作医疗覆盖全市农村。广播电视村村通全面实现。全面完成农村和县城电网改造，丘陵山区沼气入户率达75%。基本完成500个以上村庄的整治，建成100个以上小康示范村。全市80%以上的适龄农民成为具有较高素质和较强就业能力的新型农民或产业工人。村民自治水平显著提高。建设最佳人居城市的条件将基本具备。将建成中国内陆最具影响力的现代化航运港口、中部地区第一个引水及水质环境工程和现代化国际航空港。城市居民人均住宅建筑面积达32平方米。建成区绿化覆盖率达40%，绿地率达35%，人均公共绿地面积9.5平方米，城市集中式生活用水水源地水质达标率达98%，城镇生活垃圾无害化处理率达100%。最适宜创业、最适宜人居的人文环境和法治环境全面优化。以橘子洲、市博物馆、图书馆和滨江音乐厅为标志的富有地域文化特色的城市建筑和湘江生态文化景观带全面建成开放。“六桥三环”和月湖、西湖公园全面建成。山水洲城的特色和魅力更加凸现。人民生活水平和质量将明显提高。城乡居民人均可支配收入分别达18900元和6750元，恩格尔系数分别下降到32%和40%。每年新增城镇就业岗位8万个以上，农村劳动力转移就业保持80万人以上。城镇职工基本养老、基本医疗和失业保险覆盖率分别达78%、70%、74%。全面普及高中阶段教育，高等教育毛入学率达50%，每万人中具备大专以上学历人口达1200人。基本解决城乡无房户问题。人民群众健康水平全面提升，多样化文化需求得到满足。一个充满生机、富有活力、公平有序、和谐安宁、生活美好的长沙必将出现在我们眼前！

　　三、开创实施“十一五”规划工作新局面

　　2006年是“十一五”规划的开局之年，是进一步巩固和发展“十五”成果，继往开来，实现新跨越的关键一年。今年经济社会发展主要预期目标是：地区生产总值增长14%；全社会固定资产投资增长 22%；单位地区生产总值能耗降低4%；社会消费品零售总额增长15.5%；地方财政收入增长18%；城乡居民人均可支配收入分别增长9%和8%；城镇登记失业率控制在5%以内；人口自然增长率控制在5‰以内。为此，我们要突出抓好以下几个方面的工作：

　　（一）继续突出第一要务，努力实现经济平稳较快发展。一是着力推进工业化和项目建设，培育品牌优势。深化“兴工强市”战略，坚持以先进制造业促进新型工业化，加快推进产业集群发展。注重发挥两个国家级开发区的龙头作用，拉长市、县区工业园区的产业链。扎实推进工业项目建设，突出抓好100个竣工重点项目的达产、100个续建重点项目的竣工、100个计划开工项目的启动，力争十大标志性工程企业产值占规模工业产值的60%以上，全年工业投入突破200亿元，工业总产值增长22%以上。继续实施“小巨人”企业计划，重点扶持50家成长性好的中小企业。加大工业技改力度，提升产业技术水平，保护自主知识产权，培育知名品牌。二是着力推进农业产业化进程，加快发展现代农业。落实农业发展银行100亿元诚信贷款，重点扶持产业基地建设和农产品加工业，培育一批年销售收入过亿元、过10亿元的农产品加工龙头企业。大力发展养殖业，加快农业标准化进程。健全农业技术推广、农产品市场、农产品质量安全和动植物病虫害防控体系，筹建动物疫病预防控制中心。积极发展劳务经济，新增转移农村劳动力5万人。三是着力扩大消费需求，积极发展现代服务业。大力发展旅游、金融、保险、物流等现代服务业，运用现代经营方式和信息技术改造传统服务业，抓好金霞物流保税中心建设，加快以五一广场、芙蓉广场为中心的城市中央商务区建设，突出中信商务中心区建设，办好农博会和港台工业博览会等重点节会。大力拓展农村消费市场，推进“万村千乡”工程，新建和改造“农家店”100家。四是着力引进战略投资者，提升招商引资水平。提高产业引资特别是工业引资比重，注重引进战略投资项目，实现合同利用外资17亿美元，确保实际到位外资10.6亿美元。扩大外经外贸规模，市属归口管理企业实现进出口总额23亿美元。加强与国外友好城市和友好经贸合作城市的交往。

　　（二）突出城乡规划建设管理，切实推进资源节约型和环境友好型社会建设。一是加强基础设施建设，完善城市功能。全年安排重点工程项目78个，总投资116.7亿元。重点抓好湘江滨水区工程、省政府和市政府配套建设工程、火车南北货站迁建和武广客运专线长沙站配套工程、环线工程等建设。加快省道207线、国道106线、319线改造和长株高速公路建设。长潭西线、醴潭高速长沙段年底全线通车。完成霞凝港二期工程建设。启动城市轻轨规划。完成水利建设投资10亿元，着力抓好湘江枢纽工程以及福安垸等城市防洪整治工程。二是统筹城乡发展，加快新农村建设。从今年起，市财政连续三年每年安排1000万元专项资金用于村镇规划。投资2亿元实施新农村建设百村环境整治示范工程。新建、改造农村公路1000公里。加强农田水利基本建设，完成塘坝扩容增蓄3000处。切实抓好小城镇的规划、建设和管理，重点扶持建设28个小城镇、创建5个窗口示范镇，每个乡镇要办好一个新农村建设示范村。三是提升城乡管理水平，优化人居环境。开展爱国卫生运动，加大社区环境和城乡结合部的整治力度，推进农村改水改厕工作。妥善解决“城中村”问题。加快城市林业生态圈、生态动物园、浏阳河风光带等建设。开展大气污染综合治理，抓好天然气配套工程。继续落实宏观调控政策，严格管理土地，节约资源能源，大力发展循环经济。创建生态示范市和全国森林城市，启动全国环保模范城市建设和清洁能源行动。创造空气清新、山水秀美、自然和谐、环境友好的城乡环境！

　　（三）突出社会全面进步，积极促进和谐长沙建设。一是着力推进平安、诚信长沙建设。全面推进依法治市，启动“五五”普法。贯彻落实信访条例，加强信访工作。抓好基层矛盾纠纷调处工作。加强对群体性事件、突发性事件的预测预防，健全应急处置机制。加强村民自治和社区自治，开展创建“民主法治示范村（社区）”活动。加大安全生产管理力度，加强重点部位、重点行业的日常监管。实施食品药品放心工程，创建全省“食品质量安全区”。加强社会信用信息系统和失信惩戒机制建设。二是加快社会各项事业发展。深化农村义务教育体制改革，从今年秋季起对农村义务教育阶段学生全部免收杂费，年内完成现存中小学危房改造，启动职业教育基地建设。建立人才开发基金。建立和完善科技成果转化与本地化的创新体系，开展创新创业活动。完善城乡居民最低生活保障制度。全面推行新型农村合作医疗改革。健全文化市场长效管理机制，加强农村文化建设。抓好报业文化产业基地、广电中心和田汉艺术中心建设，推进铜官窑保护开发工程，加快清水塘文化旅游街区建设。大力发展原创性动漫，抓好影视文艺创作。创新人口与计划生育工作机制，稳定低生育水平，提高出生人口素质。抓好老龄工作，完善老年人活动设施和场地。广泛开展全民健身运动，备战省十运会和全国第六届城运会，完成贺龙体校建设。搞好第二次农业普查。三是认真办好八件实事。在完成省八件实事的同时，重点抓好就业再就业、农村基础设施建设、农村医疗卫生事业、社会救济扶助工作、农村义务教育和计划生育奖励、经济适用房建设和站厕改造、加强社会公共安全以及人居环境建设等市八件实事，切实解决好人民群众最关心、最直接、最现实的问题！

　　（四）突出行政管理创新，建设服务政府、责任政府和法治政府。一是改善管理与服务，建设服务政府。继续深化行政审批制度改革，加快电子政府建设，拓展政务公开的广度和深度，努力提高政务效率。进一步转变政府职能，在抓好经济调节和市场监管的同时，完善社会管理体制和公共服务体系，使政府真正成为优质公共产品的供给者、良好发展环境的创造者、人民群众利益的忠实维护者。继续深化机构改革，优化政府组织结构和人员结构，努力做到施政科学、服务到位、行为规范。二是忠实履行职责，建设责任政府。继续加强政府制度建设，推行服务承诺制、行政问责制和责任追究制，逐步形成工作目标明确、权力义务清晰、责任追究到位的行政行为责任体系。完善公共财政体系，保障公共产品的生产和供给，逐步加大财政对就业和社会保障、教育、医疗、环境保护、公共安全等方面的投入，切实做好对困难群众和弱势群体的帮扶解困工作，解决好事关群众切身利益的突出问题。全面提升政府诚信，增强政府公信力。三是严格依法行政，建设法治政府。认真实施公务员法，加强公务员队伍建设。贯彻落实行政许可法和全面推进依法行政实施纲要，深化行政执法体制改革，严格依法决策、依法办事、依法管理，树立法治政府形象。加强政风行风建设，强化行政监察、审计监督和社会监督。注重提高政府工作人员的思想道德情操修养，做到不以权谋私，不以嬉丧志，不以情枉法，不以奢毁业。各级政府公务员要志存高远，胸怀坦荡，甘于奉献，荣辱不惊，始终坚持以勤治学，以德谋政，以诚待人，以廉立身，以心为民，以更加清廉的政风带好民风，弘扬社会新风，以更加务实的作风服务群众，创造新的业绩！
